# A rare case of fibromuscular dysplasia with multifocal coronary artery involvement evaluated by intravascular ultrasound

**DOI:** 10.1016/j.jccase.2022.09.011

**Published:** 2022-10-05

**Authors:** Yuya Sakuma, Kazuhiko Nakazato, Takeshi Shimizu, Ayano Ikeda, Himika Ohara, Atsushi Kobayashi, Takayoshi Yamaki, Takafumi Ishida, Yasuchika Takeishi

**Affiliations:** Department of Cardiovascular Medicine, Fukushima Medical University, Fukushima, Japan

**Keywords:** Fibromuscular dysplasia, Coronary artery disease, Percutaneous coronary intervention, Intravascular ultrasound

## Abstract

Fibromuscular dysplasia (FMD) is non-atherosclerotic, non-inflammatory vascular disease that results in arterial stenosis. The lesions in FMD are commonly found in the renal and extracranial carotid and vertebral arteries, but the prevalence of FMD with lesions in the coronary artery is unclear. Although the vascular morphology of coronary artery lesion in FMD is mostly dissection, the following case of FMD showed the stenotic and aneurysmal lesions in coronary arteries, which was treated by percutaneous coronary angioplasty. Several vascular imaging modalities including computed tomographic angiography and catheter angiography are used for diagnosing FMD, however, the intravascular ultrasound (IVUS) imaging of the coronary artery in FMD has not been well studied. Here we describe a rare case of FMD involving multifocal coronary artery lesions with coronary aneurysm which was evaluated by IVUS imaging.

**Learning objective:**

The vascular morphologies of coronary artery lesion in fibromuscular dysplasia (FMD) mostly appear as coronary dissection, however, multifocal stenotic and aneurysmal lesions can occur in coronary arteries in FMD as the following case shows. The intravascular ultrasound findings of the stenotic coronary lesions in FMD, that were circumferential thickening of intima with various echo patterns and echolucent circumferential thickened media, may help in the diagnosis of FMD involving coronary arteries.

## Introduction

Fibromuscular dysplasia (FMD) is non-atherosclerotic, non-inflammatory vascular disease that results in arterial stenosis. The renal, extracranial carotid, and vertebral arteries are the most commonly affected in FMD. Coronary involvement in FMD is thought to be rare, but its true prevalence is unclear [1]. The vascular morphology of FMD is generally classified into two groups by the angiographic appearance; focal and multifocal [2]. The vascular morphology of coronary involvement in FMD is almost dissection and rarely appears as the multifocal lesions which are commonly found in renal arteries and carotid arteries [3]. Although several vascular imaging modalities including computed tomographic (CT) angiography, contrast-enhanced magnetic resonance angiography, and catheter angiography play important roles for diagnosing FMD, the intravascular ultrasound (IVUS) imaging of FMD has not been well studied. Here we describe a rare case of FMD involving multifocal coronary artery lesions with coronary aneurysm which was evaluated by IVUS images.

## Case report

**Fig. 1:**
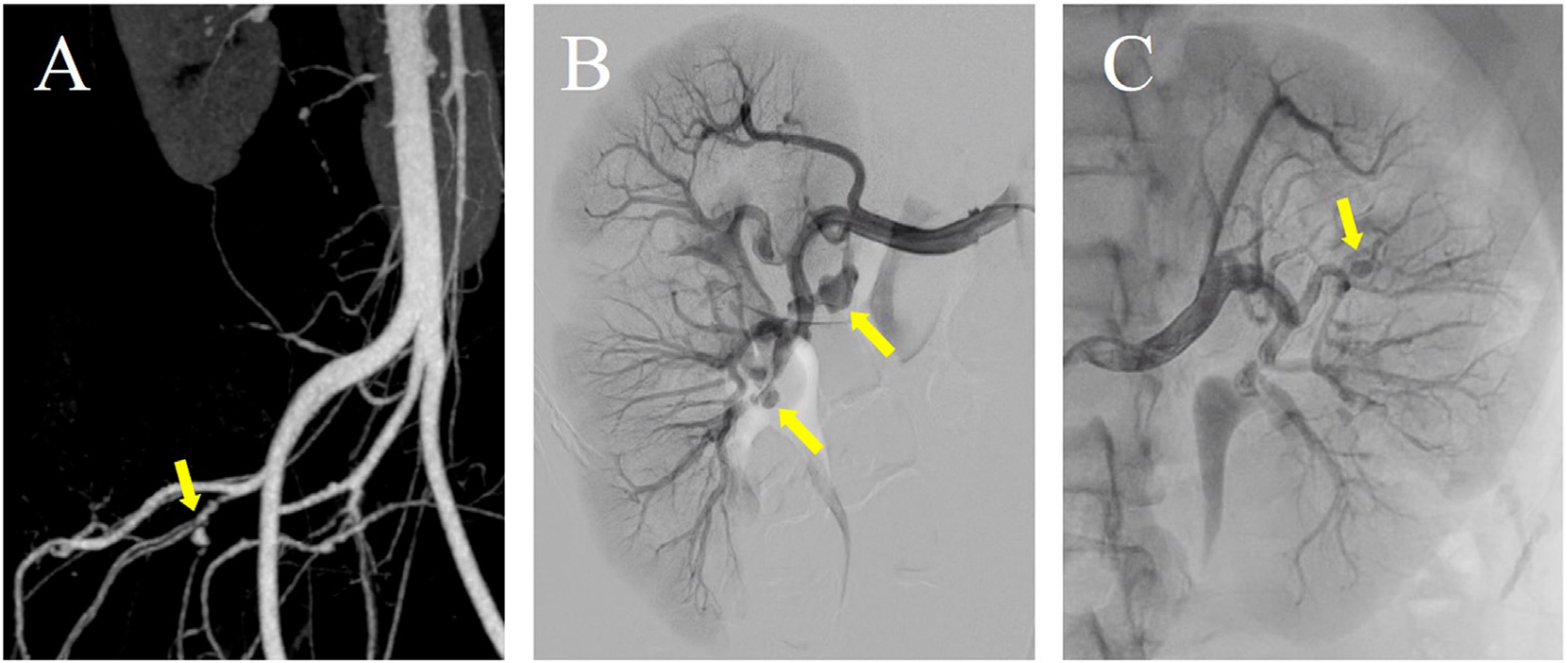
Computed tomography angiography, showing a focal stenosis in the left internal iliac artery (arrow) (A). Selective renal angiography, showing multiple stenosis and post stenotic dilatations (arrows) at distal sites of the right (B) and left (C) renal arteries.

**Fig. 2:**
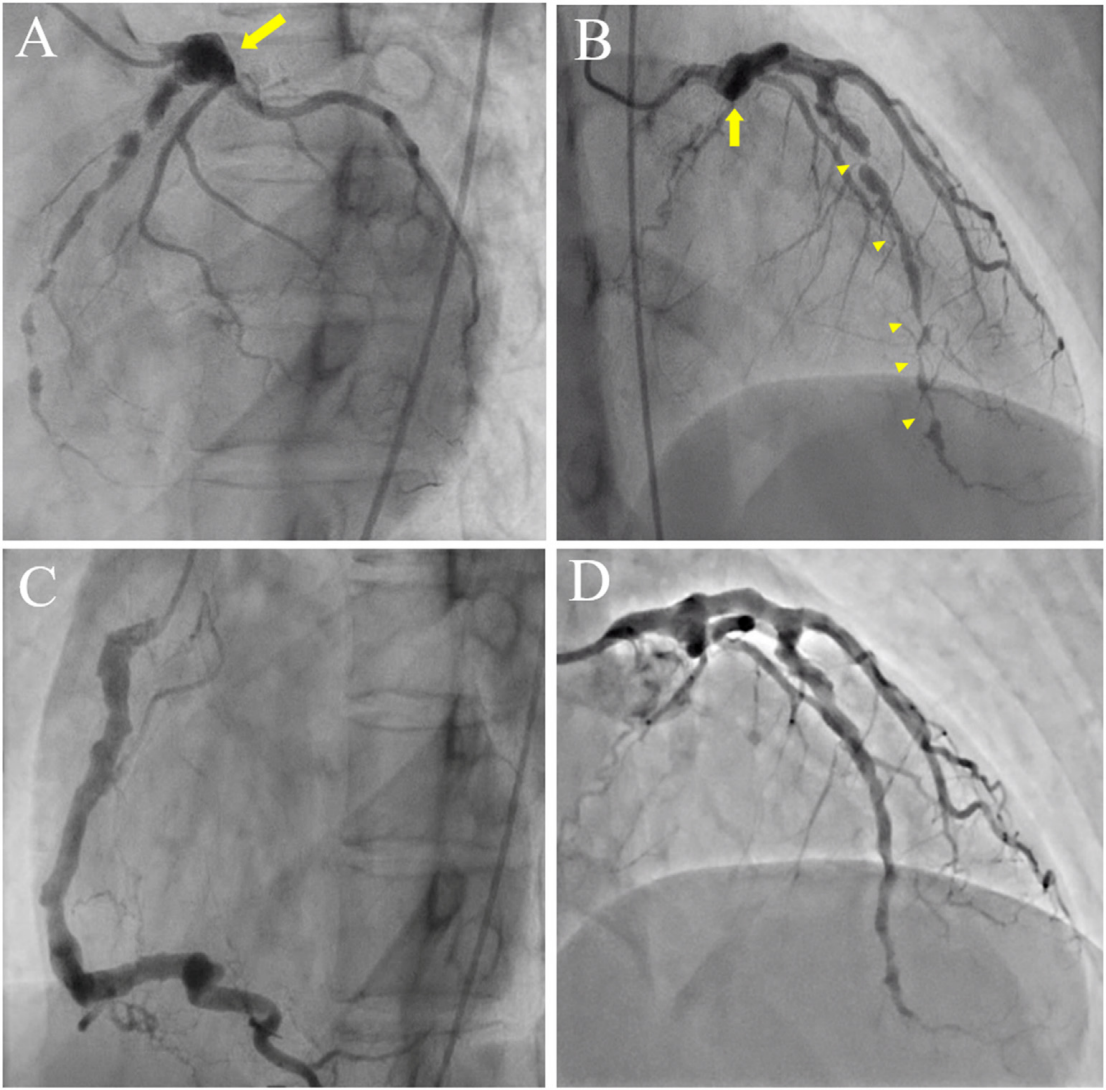
Coronary angiography, showing aneurysmal formation (arrows) of the proximal left anterior descending artery (LAD) and multiple stenotic and dilated lesions (arrowheads) in mid to distal LAD (A and B). The right coronary artery was diffusely ectasic and the atrioventricular node branch was totally occluded (C). The stenotic lesions were dilated by percutaneous coronary intervention (D).

A 26-year-old woman who had a past history of childhood asthma and atopic dermatitis presented with a chest pain radiating into left shoulder and left upper limb. She had no history of Kawasaki disease. Her family history revealed that her grandmother suffered from hypertension, aortic regurgitation with aortic root dilatation, and coronary artery disease. Her blood pressure was as high as 160/90 mm Hg. Electrocardiogram showed regular sinus rhythm and left ventricular hypertrophy. Echocardiogram revealed basal posterior wall akinesis and reduced left ventricular ejection fraction. Laboratory examination showed elevated levels of renin activity (22.6 ng/ml/h), brain natriuretic peptide (240.4 pg/ml), and troponin I (0.77 ng/ml), and normal levels of creatinine, C-reactive protein, blood glucose, and low-density lipoprotein cholesterol. There were no elevations of autoantibodies related to arteritis. The bilateral renal artery stenoses were observed by CT angiography, which also demonstrated a focal stenosis in the left internal iliac artery (Fig. 1A). The selective renal angiography showed multiple stenoses and post stenotic dilatations at distal sites of bilateral renal arteries, which seemed to be unsuitable for endovascular therapy (Fig. 1B and C). The coronary angiography revealed aneurysmal formation of the proximal left anterior descending artery (LAD) and multiple stenotic and dilated lesions of LAD (Fig. 2A and B), and the total occlusion of atrioventricular node branch of the right coronary artery (Fig. 2C). The cerebrovascular MR angiography showed no stenotic and aneurysmal formations. Based on the above findings, the patient was diagnosed as having renal hypertension due to FMD involving the internal iliac artery and the coronary arteries, which manifested the multifocal stenotic and dilated lesions.

**Fig. 3:**
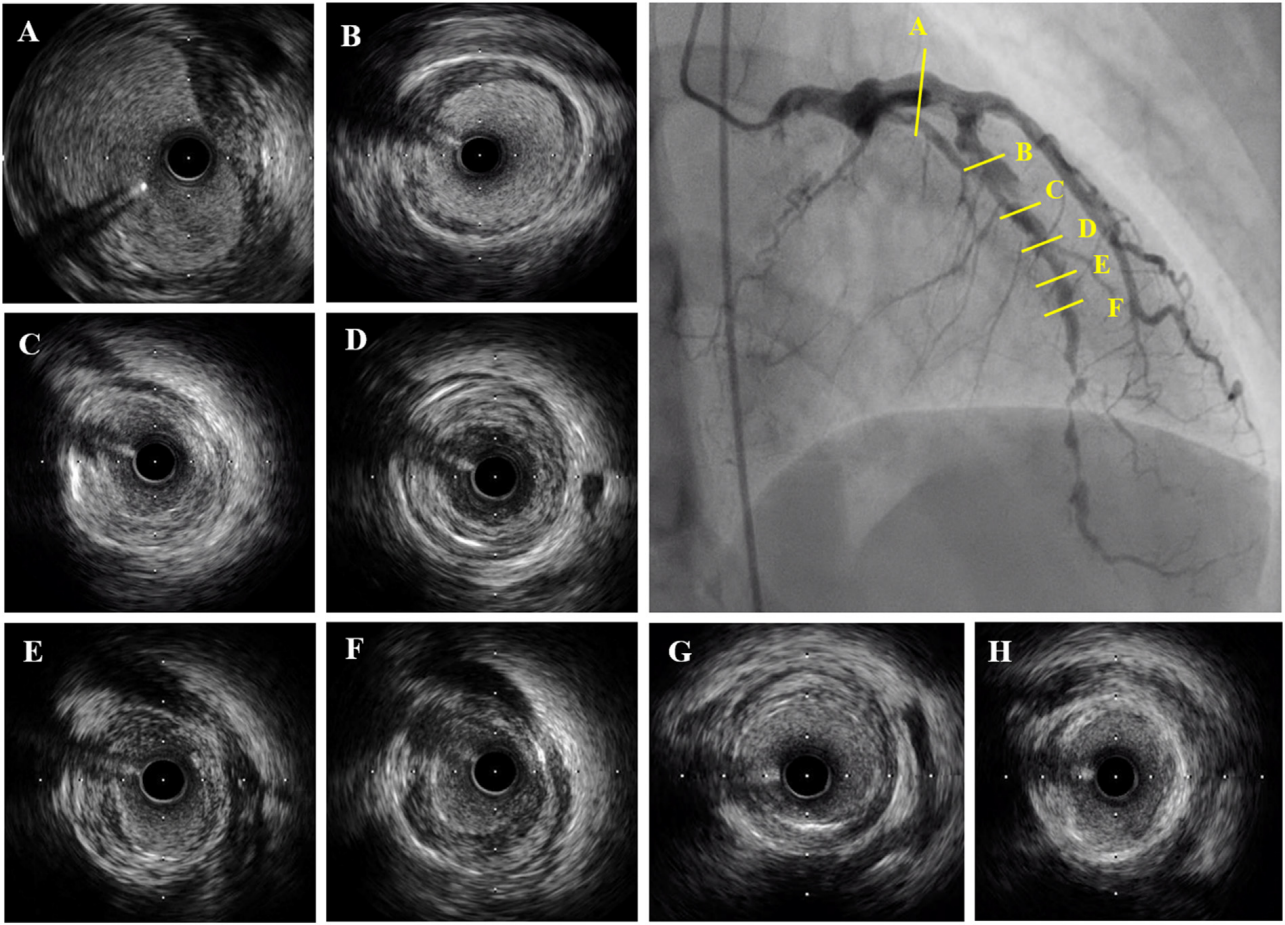
Findings of intravascular ultrasound (IVUS) in left anterior descending artery (LAD). Proximal LAD revealed aneurysmal vessel enlargement (A). The IVUS images of the stenotic lesion showed lumen narrowing by circumferential thickening of intima with various echo intensity patterns; high intensity pattern (C and D) and relatively low echo intensity pattern (E and F). Lesion in proximal segment showed echolucent circumferential band inside the external elastic membrane which might indicate thickened media (B). There were no findings of intramural hematoma or spontaneous dissection. Adequate dilation was obtained after balloon angioplasty. G and H showed the IVUS image of same lesions at D and E after balloon dilation, respectively.

Percutaneous coronary intervention (PCI) for LAD was then performed. The IVUS images of the stenotic lesion revealed lumen narrowing by circumferential thickening of intima with various echo intensity patterns; high intensity pattern (Fig. 3C and D) and relatively low echo intensity pattern (Fig. 3E and F). Lesion in proximal segment showed echolucent circumferential band inside the external elastic membrane which might indicate thickened media (Fig. 3B). The image of proximal LAD revealed aneurysmal vessel enlargement (Fig. 3A). There were no findings of intramural hematoma or spontaneous dissection. The stenotic lesions were dilated with scoring balloon (ScoreFlex® NC, OrbusNeich Medical K.K., Tokyo, Japan); 2.0 mm-diameter balloon for distal LAD and 3.5 mm-diameter balloon for proximal to mid LAD. The drug-coated balloon was not used, since the worsening aneurysmal formation was concerned. Adequate dilation and blood flow in LAD was obtained (Figs. 2D, 3G, and H). Her chest pain disappeared after the PCI and she had started to take medications including anticoagulant, β blocker, mineralocorticoid receptor antagonist, and statin. At present, no recurrence event has occurred within five months after PCI.

## Discussion

FMD is non-atherosclerotic, non-inflammatory vascular disease that may result in arterial stenosis, occlusion, aneurysm, or dissection. Lesions in FMD are commonly found in the renal and extracranial carotid and vertebral arteries, but nearly all arterial beds may be affected, and multivessel involvement is common. Approximately 80–90 % of patients with FMD are women [1]. Although the etiology of FMD is unclear, it has been supposed that hormonal, genetic, metabolic, and traumatic factors might have important roles. Gender difference in the prevalence of FMD suggests some hormonal etiologies, but there is no confident evidence. Pathological classification of FMD was proposed by McCormack et al. [2], which was based on the involved arterial layer; the intimal, medial, and adventitial FMD. A previous report has revealed that intimal FMD and adventitial FMD account for 1–2 % and <1 % of FMD, respectively. More than 90 % of all cases with FMD have been classified as medial FMD, which includes medial fibroplasia (60–70 %), perimedial fibroplasia (15–25 %), and medial hyperplasia (5–15 %) [1]. However, histological classification of FMD with collected samples for diagnosis is no longer used in modern clinical practice due to the development of endovascular therapy. Morphologically, FMD is classified into two types by angiographic appearance: (1) multifocal FMD, alternating areas of stenosis and dilatation (so called “string of beads”), which generally occurs in the mid and distal potions of the artery; or (2) focal FMD, which may occur in any part of the artery [1].

Although FMD with coronary arteries is thought to be rare, its true prevalence is unclear. In the US registry, 6.5 % of the enrolled FMD patients had histories of any coronary artery disease including atherosclerotic disease and 3.1 % had that of myocardial infarction at the time of enrollment [4]. Angiographic features of FMD involving coronary artery are mainly divided into four categories, which include spontaneous coronary artery dissection (SCAD), smooth narrowing or distal tapering typically with intramural hematoma, spasm, and tortuosity [3]. The SCAD is the most common presentation in coronary arteries with FMD, while Saw et al. reported coronary angiographic findings excluding dissected arterial segments [5]. In that report, the observed angiographic abnormalities included irregular stenosis in 59 %, smooth stenosis in 19 %, dilatation in 53 %, and ectasia in 3 %, although the multiple stenotic and dilated lesion, as in the present case, was not described. Arterial aneurysm is also the characteristic morphology in FMD, which occurs in 21.7 % of all patients with FMD. However, there was no case with a coronary aneurysm in the US registry [6] and only one case described as ectasia in the report of Saw et al. [5]. The patient in the present report showed an extremely rare manifestation that had multiple stenotic and dilated lesions and aneurysmal formation in the coronary artery.

The findings in intravascular imaging of coronary FMD are limited. Ogawa et al. previously reported that IVUS images of the renal artery stenosis with focal FMD revealed the segmental intimal-medial thickening with fibrosis appearing as echogenic pattern, and probable hyperplasia of smooth muscle cells accounting for the echolucent layer [7]. Saw et al. reported the IVUS images of coronary FMD, which included the case of SCAD with intramural hematoma, and the case of focal stenosis with intimal fibromuscular ridge and thickened medial echolucent band [8]. The dominant histologic subtype of coronary FMD is uncertain; while the most common histologic subtype in extra-coronary FMD is known as medial-type, some reports showed that intimal fibroplasia might occur often in coronary FMD [9]. In the present case, the IVUS images at coronary stenotic lesions showed circumferential thickening of intima with various echo intensity (Fig. 3C–F). The various echo intensity may represent varying degrees of collagen and smooth muscle cell accumulation. Echolucent circumferential band inside the external elastic membrane at the proximal segment indicates thickened media (Fig. 3B). Furthermore, the IVUS image at proximal LAD showed aneurysmal vessel dilatation with partially thinned medial band. To the best of our knowledge, this is the first report that showed the IVUS images of multifocal coronary lesions and coronary aneurysm in a FMD patient. These findings may help in the diagnosis of FMD involving coronary arteries.

Although the revascularization of renal artery FMD by percutaneous transluminal balloon angioplasty have been recommended for patients with hypertension, the efficacy of endovascular revascularization for coronary FMD remains unclear. The patient in this case underwent PCI since diffusely stenosed lesions reaching to distal LAD hampered coronary artery bypass grafting. The drug-coated balloon has showed its effectiveness for de novo coronary artery disease in place of stent implantation, while positive remodeling of the treated vessel has been demonstrated [10]. The lesions in this case were treated with scoring balloon but drug-coated balloon was not applied, since we were afraid of the possibility of aneurysmal formation. While there was no definitive antithrombotic therapy for coronary FMD, anticoagulation therapy was applied in this case, since no stent was used and ectasic change of the coronary artery was significant.

## Conclusions

We describe a rare case of FMD involving multifocal coronary artery lesions with coronary aneurysm which was evaluated by IVUS images. The IVUS images of coronary arteries may help in diagnosis of FMD with coronary arteries.

## Conflict of interest

The authors declare that there is no conflict of interest.
